# Losartan Attenuates Myocardial Endothelial-To-Mesenchymal Transition in Spontaneous Hypertensive Rats via Inhibiting TGF-β/Smad Signaling

**DOI:** 10.1371/journal.pone.0155730

**Published:** 2016-05-13

**Authors:** Miao Wu, Zhenyu Peng, Changhao Zu, Jing Ma, Shijuan Lu, Jianghua Zhong, Saidan Zhang

**Affiliations:** 1 Department of Cardiology, Xiangya Hospital, Central South University, Xiangya Road No.88, Changsha, P.R. China; 2 Department of Cardiology, Haikou People’s Hospital, People’s Road No.43, Haikou, Hainan, P. R. China; 3 Department of Emergency, Second Xiangya Hospital, Central South University, Middle Ren-Min Road No. 139, Changsha, P.R. China; Max-Delbrück Center for Molecular Medicine (MDC), GERMANY

## Abstract

**Background:**

Losartan plays an important role in the inhibition of myocardial fibrosis. But the underlying mechanism is not entirely clear. Emerging evidences have indicated that endothelial-to-mesenchymal transition (EndMT) plays a crucial role in cardiac fibrosis. Here the present study aims to first investigated the effect of Losartan on EndMT in cardiac fibrosis of spontaneous hypertensive rats (SHRs).

**Methods:**

Male SHRs were randomly divided into three groups and fed for 12 weeks, namely the SHR group (Group S), the Losartan-treated group (Group L) and the Prazosin-treated group (Group P). Wistar-Kyoto rats served as controls (Group W). The histological changes were evaluated by Masson’s trichrome. Co-expression of CD31 and fibroblast-specific protein 1 (FSP1) were used as the markers of EndMT through immunofluorescence. The expressions of FSP1, CD31, TGF-β, Smad were detected by Western blot analysis.

**Results:**

It was identified that elevated blood pressure induced a significant increase in myocardial fibrosis and EndMT in SHRs, which was reversed by Losartan and Prazosin treatment. Furthermore, the activity of TGF-β/Smad signaling was detected in the four groups. TGF-β/Smad signaling was activated in SHRs and suppressed by Losartan or Prazosin treatment. Losartan exhibited more efficiently than Prazosin in inhibiting TGF-β/Smad signaling activation, EndMT and myocardial fibrosis.

**Conclusion:**

These results showed that EndMT played an important role in promoting hypertensive cardiac fibrosis, and that losartan could suppress cardiac fibrosis through the inhibition of EndMT via classical TGF-β/Smad pathway.

## Introduction

Hypertension is an important health care burden worldwide. Heart failure is the most common complication seen in patients with hypertension. The major mechanism of heart failure is cardiac fibrosis, which is presented by increased stiffness and diastolic dysfunction[1]. Numerous studies have demonstrated that renin-angiotensin system (RAS) plays a crucial role in the perplexed process[2]. Angiotensin II (AngII), the major effector of RAS, is a critical profibrotic factor. AngII-receptor blockers (ARBs) could significantly decrease myocardial fibrosis, which was confirmed by many studies *in vivo* and *in vitro*[3, 4]. However, the mechanism of AngII in the progression of hypertensive cardiac fibrosis still needs further elucidation.

Hypertensive cardiac fibrosis is characterized by excessive accumulation of collagen in cardiac extracellular matrix[5]. Cardiac fibroblasts are the primary producers of collagen and have an important role in fibrosis of hypertensive heart. However, the source of fibroblasts has been debated for decades[6]. Traditionally, the main sources of cardiac fibroblasts are considered to be proliferation of resident fibroblasts and migration of bone marrow-derived circulating fibrocytes. But recent studies have indicated that Endothelial Mesenchymal Transition (EndMT) is another source of cardiac fibroblasts[7–10]. During EndMT, endothelial cells lose their specific phenotypes (such as E-cadherin, ZO-1, CD31, etc.) and gain the characterstics of fibroblasts (such as FSP1, α-SMA, vimentin, etc.). Finally, these cells convert into activated fibroblasts and migrate to the interstitial tissues through breakdown basement membranes[11–13]. Zeisberg *et al*. first verified that pressure overload promoted cardiac fibrosis through EndMT in an aortic constriction hypertensive rat model[14]. After that, EndMT was also found in animal models with diabetic cardiomyopathy, myocardial infarction and myocarditis[15–18]. However, to the authors’ knowledge, there are no researches that focus on EndMT in spontaneously hypertensive rats (SHRs). Losartan is a classic selective AT1 receptor antagonist. The antifibrotic effect of losartan has been described in many fibrotic processes such as cardiac fibrosis[19]. Although EndMT is an important factor in promoting cardiac fibrosis, it requires further study to verify whether losartan reverses fibrosis by inhibiting EndMT.

Based on aforementioned findings, the authors propose that losartan may decrease cardiac fibrosis in essential hypertension through inhibiting EndMT. In the present study, we investigated the effect of the losartan on EndMT in cardiac fibrosis using SHRs model, and tried to evaluate the role of the classic profibrotic TGF-β/Smad pathway.

## Materials and Methods

### Animals and experimental protocols

Eight-week-old male SHRs and Wistar-Kyoto rats (WKYs) were purchased from SJA Lab Animal Co. Ltd, China. After 1 week of adaptation, the SHRs were randomly divided into 3 groups and fed for 12 weeks: SHR group (Group S, *n* = 10): 1 mL saline/day by gavage; losartan-treated group (Group L, *n* = 10): 20 mg/kg/day by gavage, Wuhan Boxing, China; prazosin-treated group (Group P, *n* = 10): 5 mg/kg/day by gavage, Shanghai Zhenzhun, China. WKYs were used as control (Group W, *n* = 10; 1ml saline/day by gavage). All animals were housed in a room at a constant temperature (25°C) and exposed to a 12-hour light-dark shift. All animals were allowed free access to standard chow and water. All animal handlings were reviewed and approved by the animal ethics committee of Central South University.

### Blood pressure measurements

The SBP and DBP were measured by a tail-cuff method with the rats under conscious condition using a noninvasive blood pressure measurement system (Chengdu TME Technology Company, China). Before the measurement, rats were placed in a holding device(37°C) for 5 min. SBP and DBP were determined 3 times blind to the randomization sequence on each time point and the mean values were used as the result. SBP, DBP and heart rate measurements were taken on a weekly basis by the same evaluator.

### Echocardiographic measurements

Echocardiogram measures in this study were obtained by an experienced operator using Philips SONOS 5500 on weeks 0, 4, 8 and 12. Rats were anesthetized with intraperitoneal 10% chloral hydrate, and were underwent transthoracic two-dimensional (2D) guided M-mode echocardiography. Two-dimensional parasternal long axis views and short axis views were obtained at the papillary muscle level. Interventricular septal thickness(IVS), left ventricular posterior wall thickness at the end of diastole(LVPWd), ejection fraction(EF) and fractional shortening(FS) were measured. According to the curve of mitral diastolic flow, peak velocity at early diastole (E), peak velocity at late diastole (A) and E/A ratio were measured. Each datum was measured for 3 times and the average was taken.

### Morphological analysis

After euthanization by sodium pentobarbital (50 mg/kg), rats were sacrificed and hearts were harvested. Cardiac sections were fixed in 4% paraformaldehyde and then embedded in paraffin. Paraffin-section slides were stained with Masson’s trichrome. Photomicrographs were analyzed in a blinded manner. Photographs of left ventricle sections cut from the posterior inferior septal of each heart were for morphometrical analysis. Collagen volume fraction(CVF) and perivascular collagen area/luminal area ratio(PVCA/LA) was were observed by use of an image analysis system (Motic MED 6.0, Xiamen, China). Assessment of CVF used the following formula: CVF = collagen area/ total area (total area is exclusive of perivascular collagen area and luminal area). Mean CVF was determined by ten separate views per heart. Perivascular fibrosis was calculated as the mean PVCA/LA ratio of five intramural Arterioles per heart.

### Immunofluorescence analysis

Frozen tissues were cut into 5-μm-thick sections and fixed in 4% Polyoxymethylene at -20°C for 10 min. After 10min×3 washes in PBS, Slides were blocked in 5% bovine serum albumin (BSA) for 1 hour at room temperature. Then Slides were incubated with appropriate dilutions of the two primary antibodies overnight. The primary antibodies were mouse anti-CD31 (1:50 dilution Abcam, England) and rabbit anti-FSP1 (1:50 dilution Abcam, England). After 10min×3 washes in PBS, slides were incubated with a mixture of two secondary antibodies for 1 hour at room temperature in the dark. The secondary antibodies are Goat Anti-Rabbit, FITC (Liankebio, China) and goat anti-mouse, DyLight 549(Liankebio, China). Nuclei were counterstained with 4,6-diamidino-2-phenylindole (BioBox, China). Staining was analyzed independently by two investigators using LEICA DM5000B. Ten visual fields per heart were analyzed for co-localization of endothelial and fibroblast markers.

### Western blot analysis

The middle segment of left ventricular was collected, cut into 1.0 x 1.0cm in size, washed clean with clear water, dried with filter paper, putted in sterile tubes and stored in liquid nitrogen quickly. Frozen tissue was homogenized in liquid nitrogen, then the homogenate was lysed in cell lysis buffer (CWBIO CW2333) and PMSF (Solarbio P0100) on ice for 30 min. After centrifugation at 12000g for 20 min at 4°C, the supernatants were collected and quantified with the bicinchoninic acid (BCA) before degenerated by mixing with Laemmli buffer (biotime P0015) at 100°C for 5 min. Equal mount of proteins were loaded and separated by 10% SDS-PAGE before being transfered to polyvinylidene difluoride (PVDF) membranes. Membranes were blocked with 5% skimmed milk for at least 1 hour at room temperature, and then incubated with specific primary antibodies against CD31 (1:1000 dilution, Abcam, ab24590, England), FSP1 (1:100 dilution, Abcam, ab27957, England), COL1 (1:1500 dilution, Abcam, ab6308, England) or glyceraldehyde-3-phosphate dehydrogenase, GAPDH (1:1000dilution, Goodhere, China) overnight at 4°C. Finally, membranes were incubated with peroxidase-conjugated goat anti-rabbit IgG (1:5000 dilution, Multiscience, GAR007, China) or anti-mouse IgG (1:5000 dilution, ZSGB-bio, ZB2305, China;) and the immunoreactive bands were visualized by potent ECL kit (multiscience, China) using BIO-RAD ChemiDOc^TM^ MP imaging system(USA). Western-blot analysis was done in 3 animals per group.

### Statistical analysis

GraphPad Prism software were used to analyze data. The measurement data were presented as mean ± s.e.m. ANOVA tests were used to compare variation between groups. In case any difference between-groups is significant, post-hoc tests for multiple testing were applied. Homogeneity of variance is measured by LSD and non-homogeneity of variance is measured by Dunnet test. Results with P<0.05 were considered to have statistically significant difference.

## Results

### Body weight, left ventricular weight index(LVI) and blood pressure of the rats

After 12 weeks of feeding, Group S presented higher SBP and DBP comparing with other three groups (P<0.05, respectively). The SBP and DBP were significantly lower in Group P and Group L than Group S, and the two groups showed no significant statistically difference (P>0.05). Group W served as control with normal blood pressure. Despite a lower body weight, the LVI [left ventricle mass(mg)/body weight(g)] of the Group S was higher than the Group W (P<0.05), and it was significantly lower by Losartan or Prazosin treatment group (P<0.05). There is no significant difference between Group L and Group P in LVI. (Table 1).

**Table 1:** Body weight, LVI, blood pressure and heart rate of the rats.

Parameters	Group W (n = 10)	Group S (n = 10)	Gourp L (n = 10)	Group P (n = 10)
Body weight(g)	358.70±10.13	314.78±11.79*	333.00±19.39*#	334.67±20.64*#
LVI(mg/g)	2.24±0.13	2.77±0.14*	2.47±0.16*#	2.49±0.20*#
SBP(mmHg)	138.2±7.0	168.5±6.9*	147.4±12.6*#	154.7±11.6*#
DBP(mmHg)	105.7±10.0	124.8±14.5*	111.0±10.0*#	109.0±9.7*#
HR(bpm)	381.90±28.52	407.80±26.52*	396.10±25.46	393.90±27.54

LVI: left ventricular index; SBP: systolic blood pressure; DBP: diastolic pressure; HR heart rate.

* P<0.05 versus Group W

# P<0.05 versus Group S.

### Echocardiographic assessment

**Fig 1:**
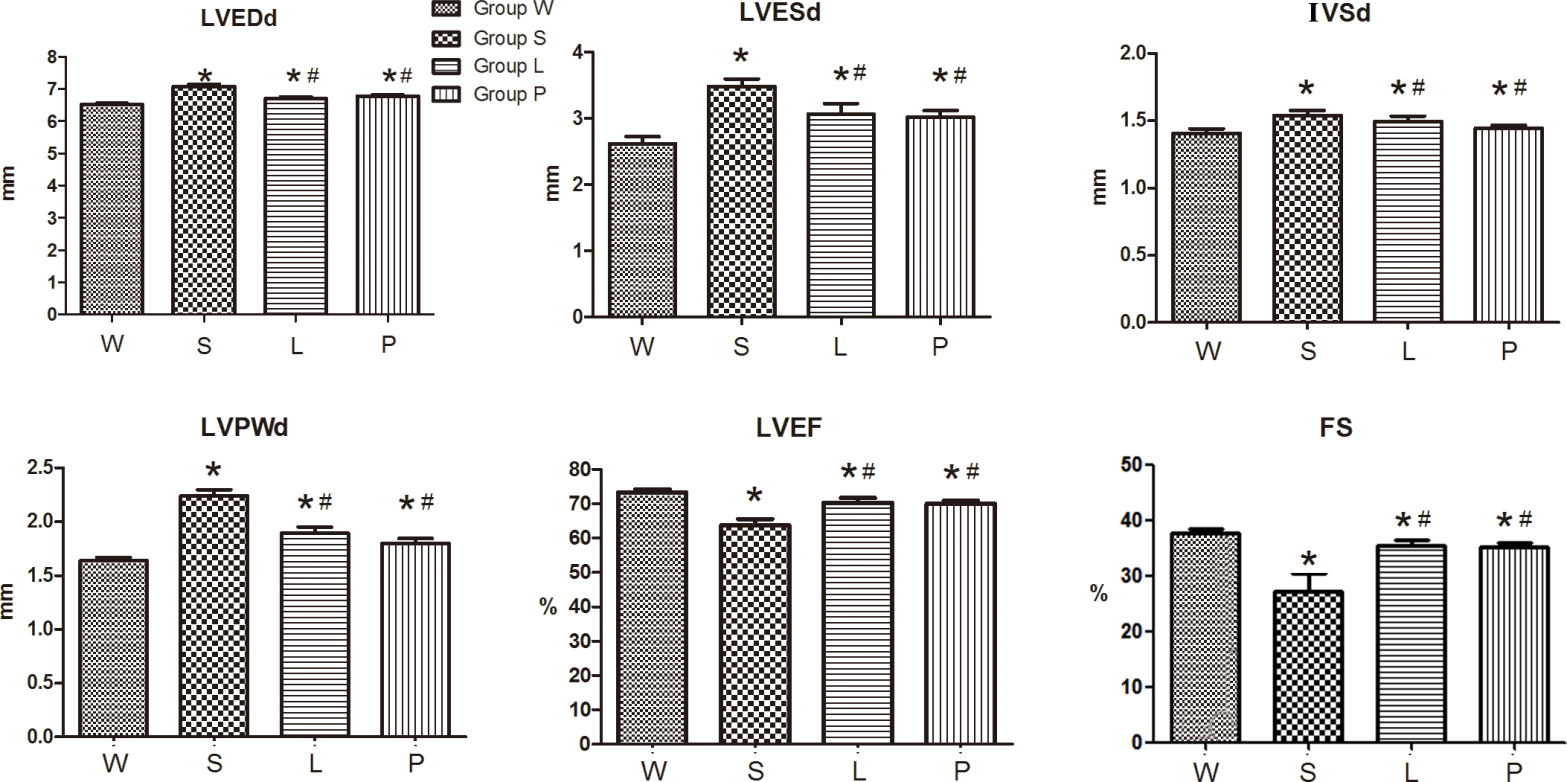
Echocardiographic parameters on the left ventricular morphology and function. LVEDd: left ventricular end diastolic internal dimension. LVESd: left ventricular end systolic internal dimension. IVSd: Interventricular septal thickness at the end of diastole, LVPWd: left ventricular posterior wall thickness at the end of diastole, LVEF: left ventricular ejection fraction. FS: fractional shortening. * P<0.05 versus Group W; # P<0.05 versus Group S

**Fig 2:**
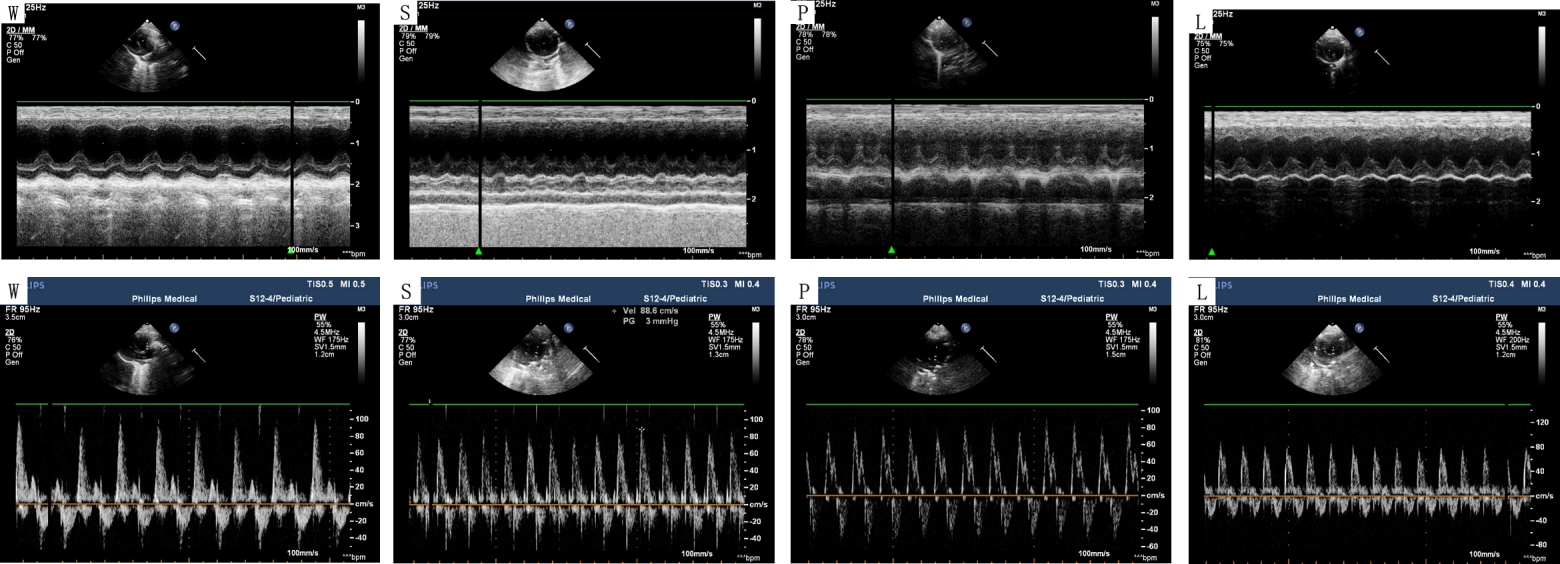
left ventricular echocardiographic representative images. LVEF and FS were significantly decreased in the Group S compared to the controls. Meanwhile, LVEF and FS were significantly increased in Group P and Group L compared to the Group S. According to the curve of mitral diastolic flow, peak velocity at early diastole (E), peak velocity at late diastole (A) and E/A ratio were measured.

After 12 weeks of feeding, the rats in Group S exhibited significantly increased levels of LVPWd, IVSd, LVESd, LVEDd and decreased levels of LVEF, FS compared to the control Group W (all P<0.05). However, LVEF and FS levels were significantly increased in Group P and Group L compared to Group S. (Fig 1, Fig 2).

### Masson trichrome

**Fig 3:**
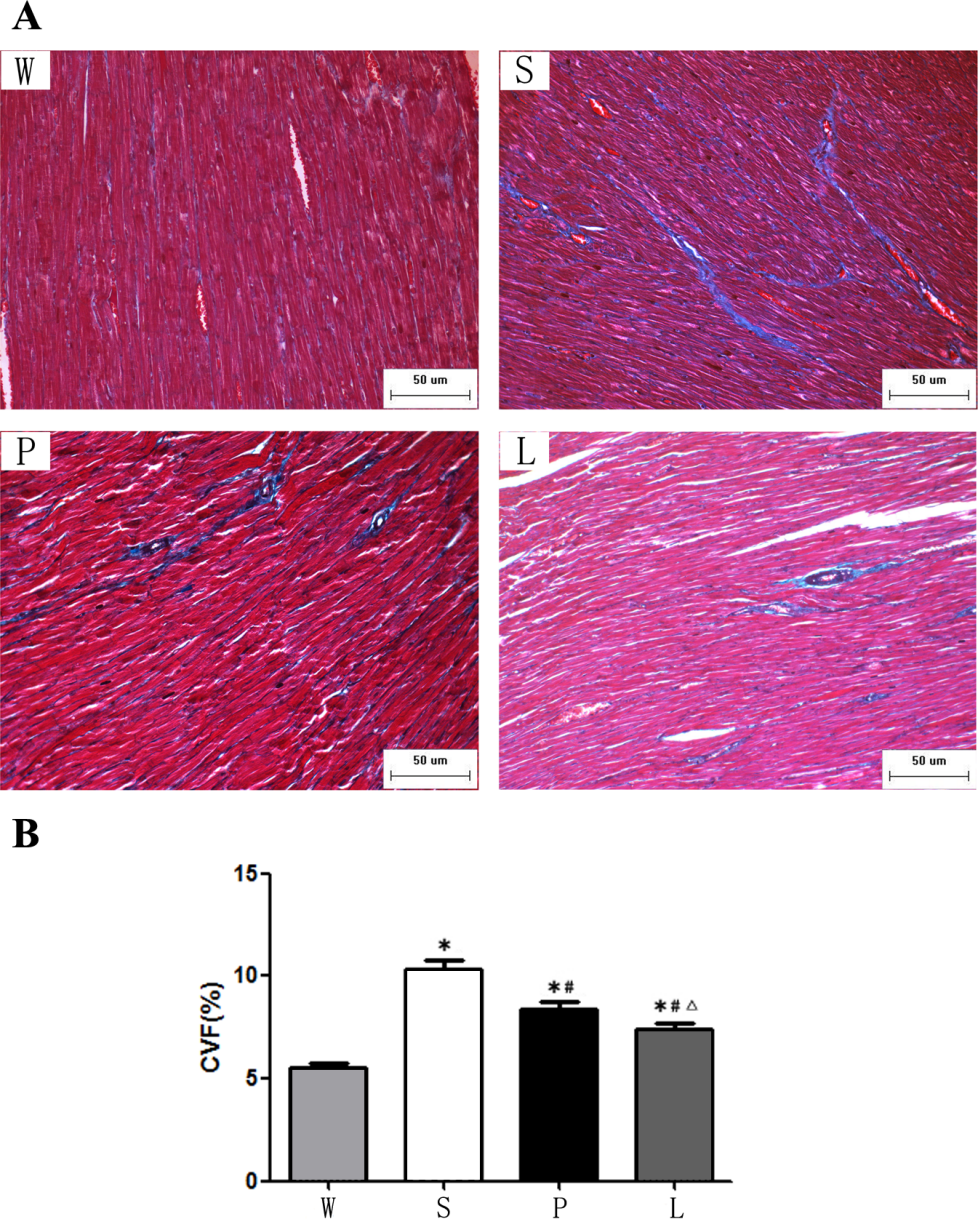
**(A)** Representative myocardial fibrosis were analyzed by Masson trichrome after 12 weeks of intervention. Scale bars, 50μm. (B) Quantification of myocardial fibrosis. * P<0.05 versus Group W; # P<0.05 versus Group S. ∆ P<0.05 versus Group P

**Fig 4:**
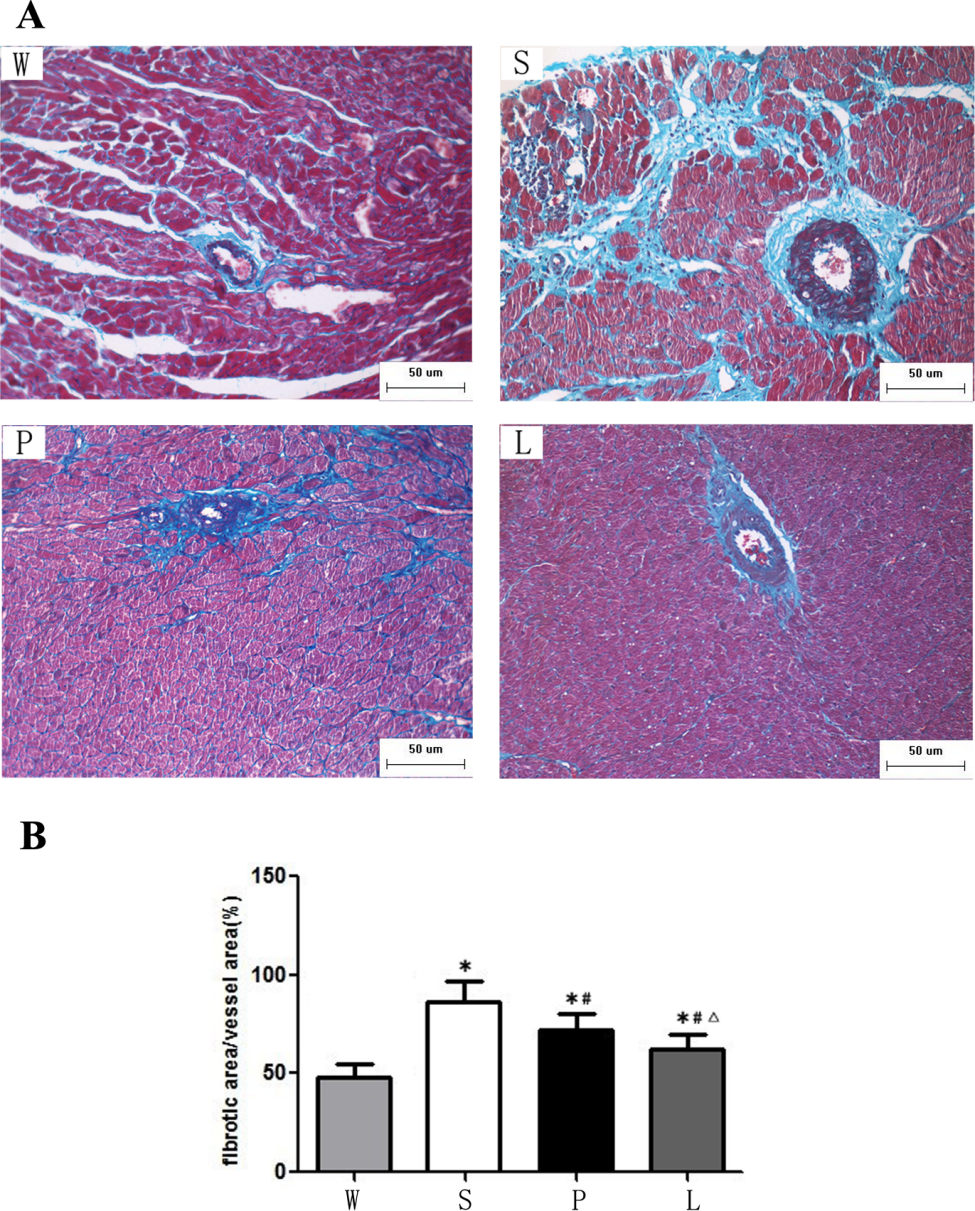
**(A)**Representative perivascular fibrosis were analyzed by Masson trichrome after 12 weeks of intervention. Scale bars, 50μm. **(B)** Quantification of perivascular fibrosis. * P<0.05 versus Group W; # P<0.05 versus Group S. ∆ P<0.05 versus Group P

Cardiac fibrosis was examined by Masson's trichrome staining. The area of myocardial fibrosis and perivascular fibrosis of Group S was significantly increased compared to the Group W while it was significantly decreased in Group L and Group P. The rats in Group L had less myocardial fibrosis and perivascular fibrosis than Group P (P < 0.05). (Fig 3, Fig 4).

### Confocal microscopy analysis

**Fig 5:**
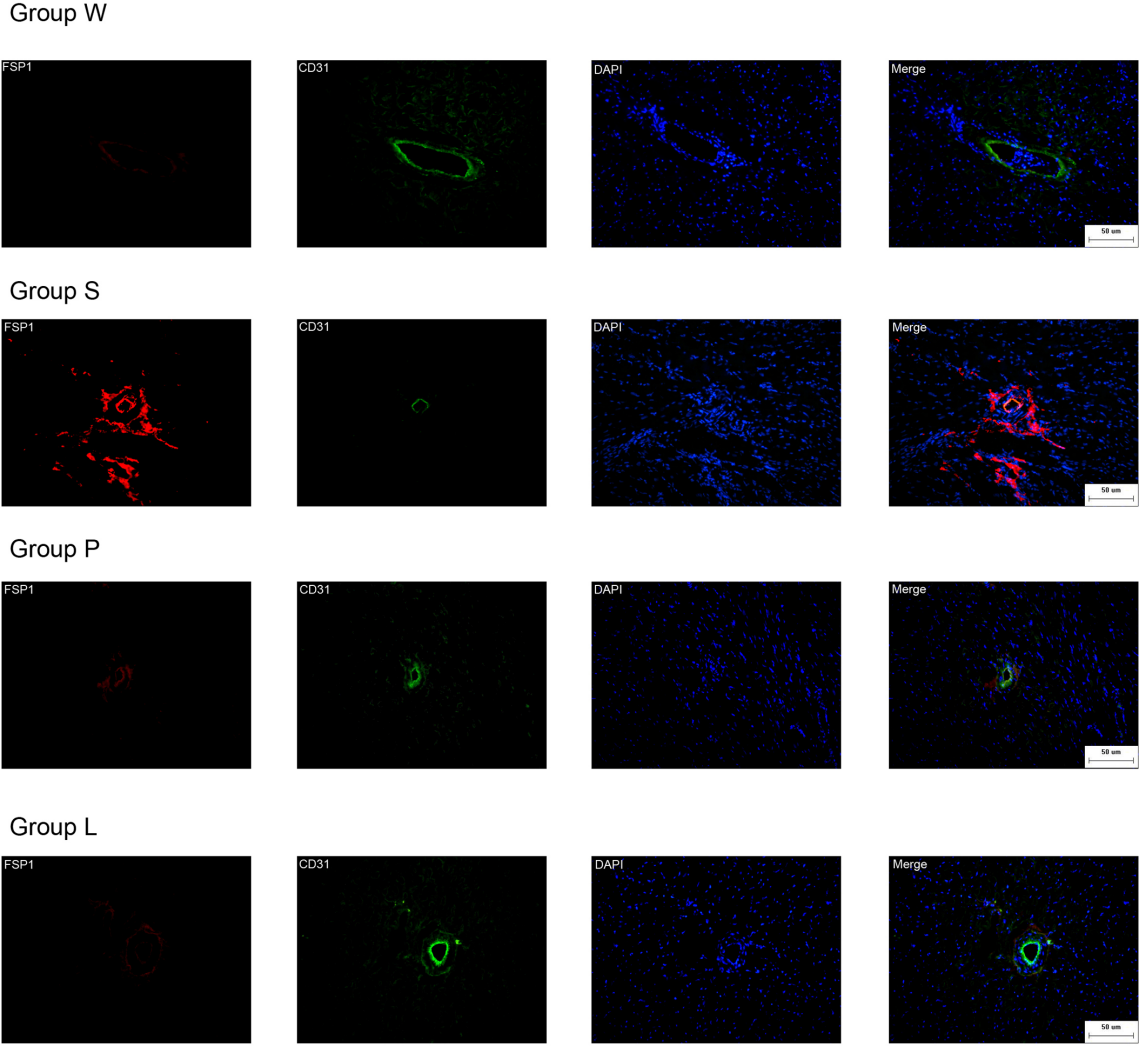
EndMT occurs in hearts of the four Groups. Confocal microscopy demonstrates FSP1 (red), CD31 (green) and DAPI (blue) in Group W, Group S, Group L and Group P. Scale bars, 50 μm.

We next investigated *in vivo* evidence of endothelial cells undergoing phenotypic transition into mesenchymal cells in hearts of the four groups. CD31 is endothelial cell marker (green) and FSP1 is fibroblast marker (red). Double labeling immunofluorescence revealed colocalization of FSP1 and CD31 (Fig 5). Double positive cells, which suggest that endothelia are beginning to acquire the fibroblast markers, an intermediate status of phenotypic conversion, are considered the evidence of the occurrence of EndMT. There is a increase of double positive cells in group S compared to group W. There were no double-positive cells in Group W. Double positive cells were found in fibrotic areas of hearts in Group S, the cells were fewer in Group P, and the fewest in Group L.

### Expression of FSP1 and CD31

**Fig 6:**
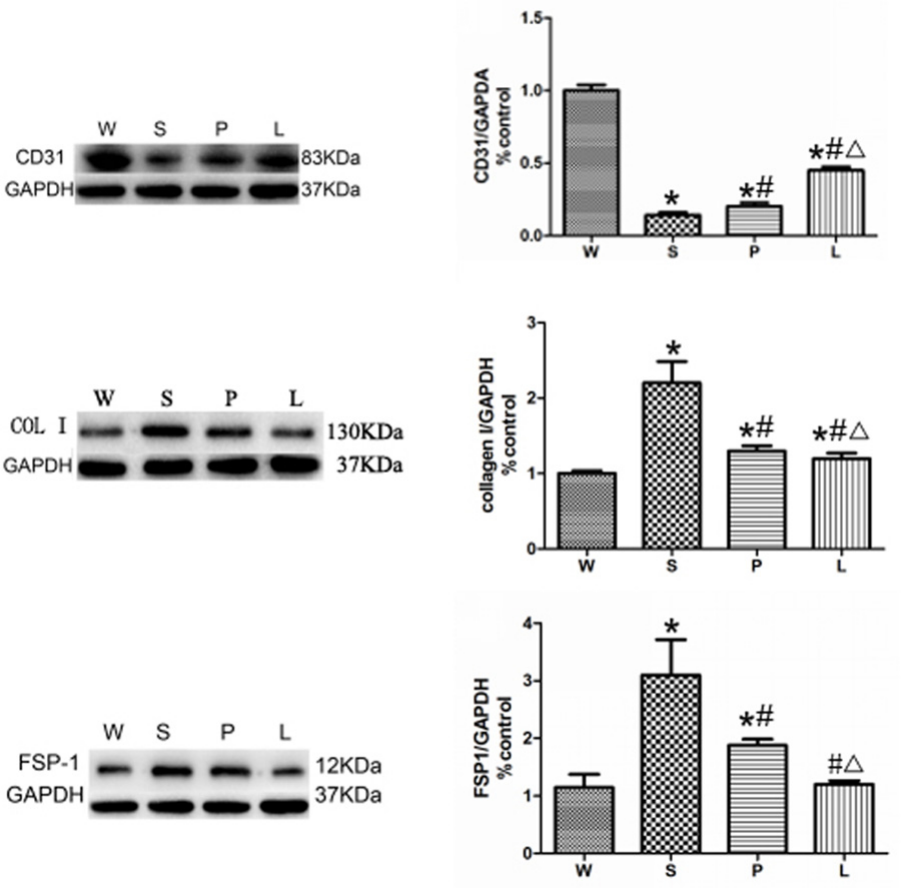
Expression ofCD31, COL I and FSP1. * P<0.05 versus Group W; # P<0.05 versus Group S. ∆ P<0.05 versus Group P.

Furthermore, the authors evaluated protein expression of FSP1, Collagen I (COL I) and CD31 by Western blot. As shown in Fig 6, the protein expression of FSP1 and COL I were markedly upregulated in the Group S comparing to Group W, and their expressions were inhibited by prazosin and losartan treatment. On the contrary, the protein expression of CD31 was significantly downregulated in the Group S compared to Group W, and their expressions were recovered by prazosin and losartan treatment. Notably, there is a higher level of CD31 and lower level of FSP1 and COL I in Group L comparing with Group P. (Fig 6)

### TGF-β/Smad signaling in hearts of the SHRs

**Fig 7:**
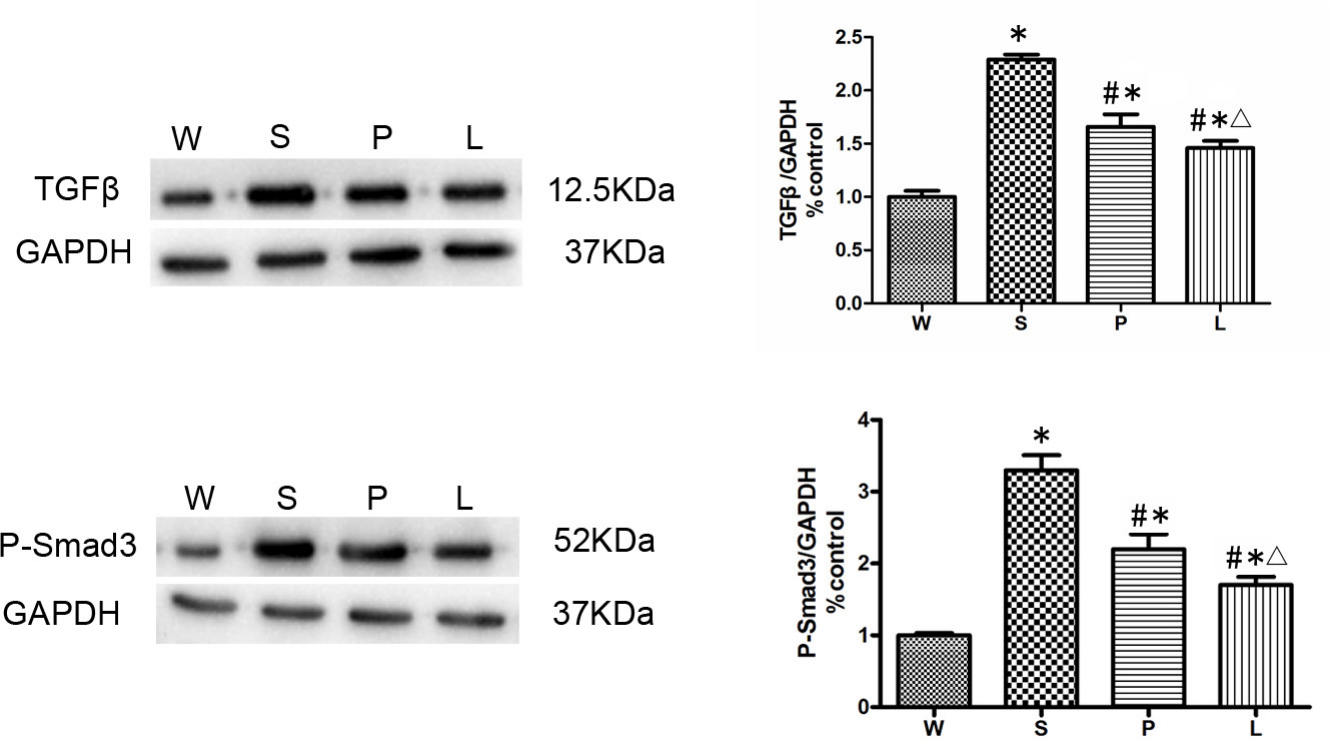
Western blot analysis for the expression of TGF-β/Smad pathway was performed on protein isolated from the hearts of the four groups. * P<0.05 versus Group W; # P<0.05 versus Group S. ∆ P<0.05 versus Group P.

Because TGF-β/Smad signaling is considered as the most common and important signaling involving in EndMT, we then evaluated the change of TGF-β/Smad signaling in the four groups using western blot after 12 weeks of intervention. Our results showed that TGF-β and phosphorylated state of Smad3 (P-Smad3) were elevated in Group S significantly comparing with other three groups (P<0.05, respectively). TGF-β/P-Smad3 decreased more significantly in Group L than in Group P (P<0.05), which indicated that losartan was more effective than prazosin in inhibiting TGF-β/Smad pathway. (Fig 7)

## Discussion

Cardiac fibrosis is an important pathological process in hypertensive heart remodeling and other heart diseases. Although cardiac fibroblasts play a key role in the remodeling of the myocardial extracellular matrix, the exact origin of fibroblasts remains ill-defined. Recently, EndMT is hotly considered as the pivotal process that converts endothelial cells to fibroblasts in heart. In that, treatments which could interfere EndMT attract a lot of attention. In this study, we demonstrated the anti-EndMT effect of losartan in SHRs by inhibiting TGF-β/Smad Signaling.

Fibroblasts are the most important fibrotic cells, but there still been many aspects needed to be clarified. The origin of cardiac fibroblasts is still not fully understood. Recent studies have confirmed that endothelial cells can contribute to the accumulation of fibroblasts in cardiac fibrosis through EndMT[20, 21]. In 2007, Zeisberg and colleagues published a milestone research of EndMT in cardiac fibrosis. The group confirmed 27% to 35% of all fibroblasts are endothelial origin using aortic banding double-transgenic rats[14]. Similarly, to our knowledge, we report for the first time that EndMT also plays an important part in cardiac fibrosis of SHRs. During EndMT, endothelial cells lose their intercellular adhesion complexes including E-cadherin, ZO-1, CD31, etc., and detach from the surrounding cells and basement membrane. In the meantime, the transformed endothelial cells acquire mesenchymal markers, such as FSP1, α-SMA, vimentin, etc. and gain the morphology and function of activated fibroblasts. In the present study, CD31 was used as a specific endothelial marker and FSP1 as mesenchymal marker. Coexpression of CD31 and FSP1 indicates that endothelial cells are undergoing EndMT. Our experiments show that WKYs have less double labeling cells, but SHRs have significant CD31+/FSP1+ cells in the three groups through double immunofluorescence staining.

Moreover, it is find the more myocardial fibrosis was, the more obvious EndMT happened. After 12 weeks of observations, untreated and losartan or prazosin treatment SHRs developed left ventricular dilation, heart dysfunction, EndMT and fibrosis, suggesting that EndMT contributes to cardiac fibrosis in SHRs. Zeiberg has confirmed EndMT in an aortic banding model in which pressure overload is abruptly increasing during a very short period. It is inconsistent with the pathophysiological mechanisms of human essential hypertension. There are many factors that can cause hypertension in SHRs, such as increasing peripheral arterial resistance, oxidative stress, RAS activation, etc. On the contrary, pressure overload via ascending aortic constriction leads to an acute increase of heart afterload, which lead to myocardial fibrosis in a short time. It is distinct from pathophysiology of human essential hypertension. Thus, we investigate the role of EndMT in SHRs which is more similar to the natural history of human essential hypertension. Our data are consistent with the results of other cardiac fibrosis models including diabetic cardiomyopathy, myocardial infarction, aortic banding and myocarditis. It may be concluded that EndMT is a common process in cardiac fibrosis despite of causes.

Previous evidence suggested that elevated blood pressure was linked to an increase of fibrosis in the heart. In our study, antihypertensive drugs reduce cardiac fibrosis and EndMT, which is consistent with the result of the aortic banding rats. Numerous studies showed that hypertensive cardiac fibrosis is related to RAS. RAS is a complex and crucial system in cardiovascular disease. AngII is the principal effector of RAS, which play an important role in cardiac fibrosis. However, there have been no researches about the role of AngII in EndMT of hypertensive cardiac fibrosis. Our results indicate losartan, an angiotensin receptor 1 inhibitor, decreases fibrosis and EndMT in SHRs. Therefore, AngII takes an essential effect in hypertensive cardiac fibrosis through promoting EndMT.

Pressure overload and RAS are two main triggers of cardiac fibrosis in hypertension[22, 23]. In most situations, it’s difficult to separate the two factors apart completely because they closely interact with each other. Prazosin, a peripherally alpha-adrenoceptor antagonist, reduces blood pressure but with no direct effect on RAS. In this study, SHRs treated with Losartan and Prazosin have similar blood pressure. Both groups have decreased cardiac fibrosis area and CD31+/FSP1+ cells comparing to untreated SHRs. But cardiac fibrosis and double-positive cells were more significantly decreased in Group L than Group P. As we know, Losartan is associated with the lowering blood pressure and anti-RAS effects, from which the stronger inhibition of EndMT comes. Therefore, the results suggest that both pressure overload and RAS contribute to EndMT in cardiac fibrosis. Losartan inhibits EndMT in hypertensive cardiac fibrosis via decreasing blood pressure and inhibiting RAS. This provides a new mechanism for the anti-fibrotic effect of ARB.

In the present study, the results suggested that losartan inhibits EndMT and fibrosis more effectively than prazosin, but this does not lead to functional improvements. This result differed from previous researches that angiotensin receptor blockers more effectively decrease fibrosis, inhibit myocardial remodeling and improve heart function than prazosin. There may be several reasons for the inconsistency. First, the duration of observation and dose of losartan may not be enough. The dose range of losartan in previous studies was very wide from 5 mg/kg/day to 50 mg/kg/day[24,25]. Differences between the two groups may occur with a higher dose or a longer observation time. Second, the techniques used to evaluate cardiac function were probably not sensitive enough. Though echocardiography is widely used to measure left ventricular function, accuracy of EF is affected by left ventricular preload, afterload, heart rate, left ventricular shape, operator, *etc*. Additional methods, such as hemodynamics measurements, tissue Doppler imaging and heart magnetic resonance imaging (MRI), will provide more accurate information of heart structure and function[26]. These are the limitations of the paper.

EndMT is regulated by a variety of bioactive molecules, including transforming growth factor-β (TGF-β), endothelin-1 (ET-1), angiotensin II (AngII), connective tissue growth factor (CTGF), and platelet-derived growth factor (PDGF)[27–29]. Numerous studies showed TGF-β/Smad is a vital signaling pathway in EndMT[30]. It's well-known that AngII activate TGF-β/Smad pathway in cardiac fibrosis[11, 31–33]. Our results demonstrated that the decreasing of EndMT is consistent with reducing signaling of TGF-β/Smad. TGF-β/Smad signaling pathway was more prominently inhibited in SHRs treated with losartan than prazosin. Altogether, these *in vivo* data suggested that the inhibition of EndMT in SHRs is mediated by losartan through inhibition of TGF-β/Smad signaling pathway.

## Conclusion

In summary, our work shows EndMT is one of the origins of fibroblasts in hypertensive cardiac fibrosis, and that losartan decreases cardiac fibrosis partly through inhibition of EndMT via classical TGF-β/Smad pathway. EndMT might be a therapeutic target to prevent cardiac fibrosis and slow down heart failure in the future.
